# Is there any role for medical thoracoscopy in the treatment of empyema in children?

**DOI:** 10.1111/crj.13578

**Published:** 2023-01-03

**Authors:** Lina Zuccatosta, Sara Piciucchi, Sabrina Martinello, Fabio Sultani, Stefano Oldani, Arianna Johanna de Grauw, Stefano Maitan, Massimo Ruggero Corso, Venerino Poletti, Claudia Ravaglia

**Affiliations:** ^1^ Pulmonology and Bronchology Unit Marche Polytechnic University Hospital Ancona Italy; ^2^ Radiology Unit G.B. Morgagni Hospital/University of Bologna Forlì Italy; ^3^ Pulmonology Unit G.B. Morgagni Hospital/University of Bologna Forlì Italy; ^4^ Intensive Care Unit G.B. Morgagni Hospital/University of Bologna Forlì Italy; ^5^ Dipartimento di Scienze Mediche e Chirurgiche (DIMES) Alma Mater Studiorum University of Bologna Bologna Italy

**Keywords:** children, empyema, medical thoracoscopy, pleural effusion, thoracoscopy

## Abstract

It is still controversial whether surgical or nonsurgical treatment approaches are most appropriate for empyema in children, and there are no data regarding the role of medical thoracoscopy in this population. The aim of this study was to describe our experience with medical thoracosocpy in children with multiloculated and organizing pneumonia. We retrospectively reviewed children admitted to our hospital with a diagnosis of empyema from 2011 to 2021 and treated with medical thoracoscopy. A total of six patients with empyema were treated by medical thoracoscopy; empyema was multiloculated in five cases and organized in one case; all children in the study recovered completely with full lung expansion after chest X‐rays, and no disease sequelae were reported after clinical follow‐up. Our small case series suggests that in selected cases, medical thoracoscopy could safely and effectively treat pleural empyema in children, with less invasiveness and reduced psychological consequences.

## Graphical abstract

This study reviewed children admitted with a diagnosis of multiloculated or organizing empyema and treated with medical thoracoscopy. All children had complete recovery with full lung expansion according to chest X‐ray and no disease sequelae. Pleural empyema in children could be treated safely and successfully by medical thoracoscopy in selected cases, resulting in less invasiveness and decreased psychological sequelae.

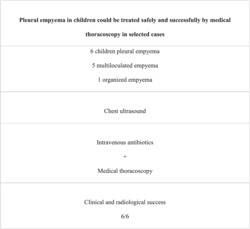

**Abbreviations**
CT: computed tomography
ICU: intensive care unit
PICU: pediatric intensive care unit
TB: tuberculosis
US: ultrasound
VATS: video‐assisted thoracoscopic surgery

## 1 INTRODUCTION

It has been reported that 5% of children hospitalized with community acquired pneumonia may develop empyema.^1^ Treatment for pediatric empyema involves antibiotics, chest drainage (with or without fibrinolytic agents), and a variety of surgical techniques for empyema resolution and lung decortication, similar to adult empyema management.^2^ The majority of pediatric practice data is derived from adult studies; however, part of the problem is the lack of pediatric trials, and extrapolating adult data to children is not appropriate. As a result, there remains controversy regarding the most suitable treatment approach for the pediatric population, namely, operative versus nonoperative,^3^ and the current literature provides no data regarding the role of medical thoracoscopy in children. This study aimed to report our experiences regarding the safety and efficacy of medical thoracoscopy in children with multiloculated and organizing empyema.

## 2 METHODS

We retrospectively evaluated all children admitted to our hospital with a diagnosis of empyema from 2011 to 2021 and treated with medical thoracoscopy (Morgagni Hospital, Forlì, Italy); pleural effusions with no signs of empyema were not considered because these were not treated by thoracoscopy. All patients underwent an ultrasound (US) with a high‐frequency linear transducer (5–7.5 MHz) and a convex transducer of intermediate frequency (3–4 MHz). Empyema was diagnosed by identifying pus during thoracentesis (turbid malodorous liquid or pH 7.2) with laboratory signs of infections; multiloculated empyema was defined as ultrasonographic presence of multiple empyema loculations with intrapleural septae, while organized empyema was characterized by the evidence of the thickening of the pleura. Unlike computed tomography (CT), US is portable, relatively inexpensive, and does not expose the patient to radiation. US can reliably differentiate between parenchymal and pleural processes and determine the cause of pleural loculations.^4^ CT scans were performed only in cases of previous radiographic and ultrasonographic findings associated with possible complex pleural disease, according to a small retrospective review comparing US and CT (which found that CT had no benefit in most cases).^5^

Medical thoracoscopy was carried out in the lateral decubitus position under local anesthesia with 2% lidocaine and moderate sedation (propofol 8 ml/h and remifentanil 0.15 mcg/kg/min) with inhalational induction (sevoflurane 8%). Trocar (of 5.5 mm in three cases and 7.5 mm in one case) was inserted under ultrasonographic guidance in the appropriate intercostals space (single access in five cases and double access in one patient with organizing empyema), and a video‐thoracoscope (Storz, Wolf, Germany) provided with a 0° optical telescope was inserted. We aspirated and removed fluid and fibrinopurulent material from the pleural cavity using closed biopsy forceps after carefully inspecting the pleura. At the end of the procedure, a drain (16–20 F) was inserted and connected to underwater seal suction with a negative pressure suction of 20 cmH_2_O. Duration of the procedure was 27 min (range 15–55 min). Patients received intravenous antibiotics for at least 1 week after the procedure, and in four children, 25 000 U of urokinase diluted in 30 ml of normal saline solution was administered into the pleural space once daily for 3 days (range 2–4 days), and after rinsing with the saline solution, the drain was clamped for 2 h.^6^

Treatment success was defined as radiologic confirmation of successful pleural drainage (i.e., reduction of the size of pleural fluid on the chest X‐ray and thoracic US of less than one third of the hemithorax in complete resolution or greater than one third in partial resolution), without the need for further treatment (subsequent chest tube insertions or surgical interventions) and objective evidence of sepsis resolution (improvement in temperature and clinical condition and decreasing inflammatory laboratory markers).

The success of treatment was determined by radiologic confirmation of successful pleural drainage without further treatment (subsequent chest tubes or surgical interventions) and objective evidence that sepsis had resolved (improved temperature and clinical condition and decreased inflammatory laboratory markers). Successful pleural drainage was characterized by reduction of the size of pleural fluid on the chest X‐ray and thoracic US to less than one third in complete resolution or greater than one third in partial resolution.

## 3 RESULTS

A total of six children with empyema were treated by medical thoracoscopy. Five patients had multiloculated empyema (phase II) and one had organizing empyema (phase III). Mean age was 6.9 years [range 5–10 years]; four were male and two were female. In four patients, a microbiological diagnosis could be made (one *Streptococcus pneumoniae* and three *Mycobacterium tuberculosis*). All children received broad spectrum antibiotic treatment after the thoracoscopy which was then targeted based on the culture results if available; the children with tuberculous empyema were also treated with anti‐tuberculosis (TB) medications. Four children received adjuvant postthoracoscopy intrapleural fibrinolysis (Urokinase) once daily for 3.3 days (range 3–4 days): one with organizing empyema and three with multiloculated empyema. Chest tube drainage after medical thoracoscopy was maintained for 3.1 days (range 2 to 6 days); we did not have a standard criterion for discontinuation of chest tube drainage, and duration of chest tubes was longer in the case with organizing effusion.

According to chest X‐rays, all children in the study recovered completely. After treatment for empyema and recovery from pneumonia, the patients were discharged. No disease sequelae were reported at clinical follow‐up. No major adverse effects of urokinase were noted, such as anaphylactoid reaction, arrhythmias, bleeding, polyneuropathy, or noncardiogenic pulmonary edema. Full lung re‐expansion was observed during an ambulatory follow‐up at least 1 month after discharge.

## 4 DISCUSSION

The treatment of pediatric empyema can differ significantly from that in adults due to the superior healing potential of pediatric patients; furthermore, despite the fact that pediatric empyema poses less of a serious threat than adult empyema, where mortality can approach 20%, it still poses a significant burden on hospitals and families. In pediatric empyema, video‐assisted thoracic surgery is considered to have certain advantages over open surgery because of its safety, efficiency, shorter hospital stays, and earlier recovery.^7^ Although video‐assisted thoracoscopic surgery (VATS) does not provide any therapeutic or recovery benefits over the nonoperative approach of fibrinolysis for pediatric empyema, recent evidence suggests that fibrinolysis may pose a lower risk of acute clinical deterioration and could be the first‐line treatment for children with empyema.^6^ As compared with the approach with chest tube drainage, no significant differences were found in the length of stay with repeated US thoracentesis.^8^ However, the latter approach may be appropriate only for older children (who can tolerate local anesthetics and sedation) and may not be a cost‐effective management strategy when considering the number of sedation procedures required.^9^ For cases in which the first thoracentesis fails to adequately drain the effusion, the British Thoracic Society recommends a chest tube. In retrospective and prospective studies, fibrinolysis has been shown to be superior to chest tube drainage alone.^2^

The results of our small case series suggest that selected cases of pleural empyema in children can be successfully treated by medical thoracoscopy; in adults, these results have already been described,^10^, ^11^ but there are no published studies on medical thoracoscopy in children.

A medical thoracoscopy is less invasive and less time‐consuming than video‐assisted thoracoscopy, because it is performed under local anesthesia and moderate sedation. With medical thoracoscopy, multiple loculations can be opened and purulent liquid can be aspirated, fibrinous adhesions can be removed, and fibrinolytics can be applied locally; this technique may have some limitations because, unlike surgical VATS, it is usually performed via a single port, so the lung is not fully collapsed during surgery. As a result of the lower number of pediatric intensive care unit (PICU) admissions among nonoperated pediatric empyema patients and the shorter stay in PICU, health facilities have also enjoyed a lighter ICU workload, which has a direct effect on both caregivers and patients. PICU stays have a significant psychological impact on children. As a result, psychological aspects should be considered when selecting a treatment approach that affects these PICU‐related outcomes, especially as less invasiveness is directly related to a decrease in psychological complications.^12^

All children in our report had complete recovery with full lung expansion according to ultrasonography and chest X‐ray. No disease sequelae or chronic morbidity were reported at clinical follow‐up. Regarding the use of intrapleural fibrinolytics, no adverse effects of urokinase were noted, and our results confirm that intrapleural urokinase could confer significant benefit in reducing the requirement for surgical intervention in empyema.

The very small sample size and retrospective nature of this series pose important limitations; results from larger samples may be more generalizable, and prospective studies may reveal additional characteristics of noninvasive empyema treatment.

## CONFLICT OF INTEREST

None of the authors have conflicts of interest.

## ETHICS STATEMENT

This study was performed in accordance with regulations issued by the Helsinki Declaration; the protocol was approved by the Institutional Review Board (IRB) of the Area Vasta Romagna Ethical Committee (project approval No. 6448/2020 I.5/140), and patients parents have provided informed consent according to the rules applied in our Hospital, accepting potential risks and advantages of the proposed procedure.

## AUTHOR CONTRIBUTIONS

LZ, VR and CR conceived and designed the study. CR and LZ wrote the paper. LZ, SP, SM, FS, SO, AJDG, SM, MRC, VP and CR took part in collecting the data. LZ, SP, VP and CR analysed the data.

## Data Availability

The data that support the findings of this study are available from the corresponding author upon reasonable request.
